# Think outside the box – atypical infections in chronic sinusitis

**DOI:** 10.18632/oncoscience.576

**Published:** 2023-05-27

**Authors:** Florian Dudde, Kai-Olaf Henkel, Filip Barbarewicz

**Affiliations:** ^1^Department of Oral and Maxillofacial Surgery, Army Hospital Hamburg, Hamburg, Germany

**Keywords:** chronic sinusitis, aspergilloma, dental treatment

Inflammations of the paranasal sinuses represent a common clinical picture. The annual prevalence of chronic sinusitis in Europe is up to 10% [1]. Sinusitis can be divided into acute and chronic forms. In particular, the chronic forms (>12 weeks duration) are often challenging in the context of therapy. In general, all ventilation disorders of the paranasal sinuses (concha bullosa, nasal septal deviations, etc.,) represent risk factors for the development of any form of sinusitis [2]. In addition, an immune deficiency or systemic diseases relevant to the immune system predispose to infections with atypical pathogens. Most sinusitis are caused by viruses, sometimes bacteria and, in rare cases, fungal infections [3]. Furthermore, sinusitis can be differentiated with regard to the affected paranasal sinuses. Unilateral sinusitis can often result from a dental focus and/or a fungal infection such as aspergillosis [4, 5]. However, pathophysiologically, active and chronic sinusitis differ in terms of the sometimes irreversible remodeling processes in the mucosa [6].

The symptoms of sinusitis are varied (increased nasal secretion, constipation, olfactory disorders, facial pain, etc.,) especially in the context of chronic sinusitis. While the diagnosis of acute sinusitis is made clinically, the clarification of chronic sinusitis requires extended imaging (e.g., Low-dose CT of the paranasal sinuses) and nasal endoscopy. Alternatively, an MRI examination and/or cone beam scan (DVT) examination of the affected region can also be carried out. If the findings are unclear, an odontogenic sinusitis needs to be discussed in the interface area of the clinical subjects of ear-nose-throat department (ENT) and the department of maxillofacial surgery.

In addition to conservative treatment options for chronic sinusitis (glucocorticoid nasal sprays, antibiotics, antimycotics, immunotherapy), surgical procedures (functional endoscopic sinus surgery) can also be considered. However, chronic sinusitis tends towards a high rate of recurrences. Therefore, in many cases only symptom control is achieved [7]. In particular, treatment-refractory forms of chronic sinusitis with unclear etiology require interdisciplinary diagnostics and treatment, as we were able to clearly demonstrate in the article recently published [8].

Fungal infections of the paranasal sinuses are rare cases. In particular, systemic diseases relevant to the immune system such as diabetes mellitus predispose to infections with atypical pathogens. Infections with Aspergillus in the area of the paranasal sinuses often only occur in a single paranasal sinus. Aspergillomas in the maxillary sinus are frequently associated with root canal treatment in the area of the upper jaw teeth due to overpressed root filling material into the maxillary sinus.

**Figure 1:**
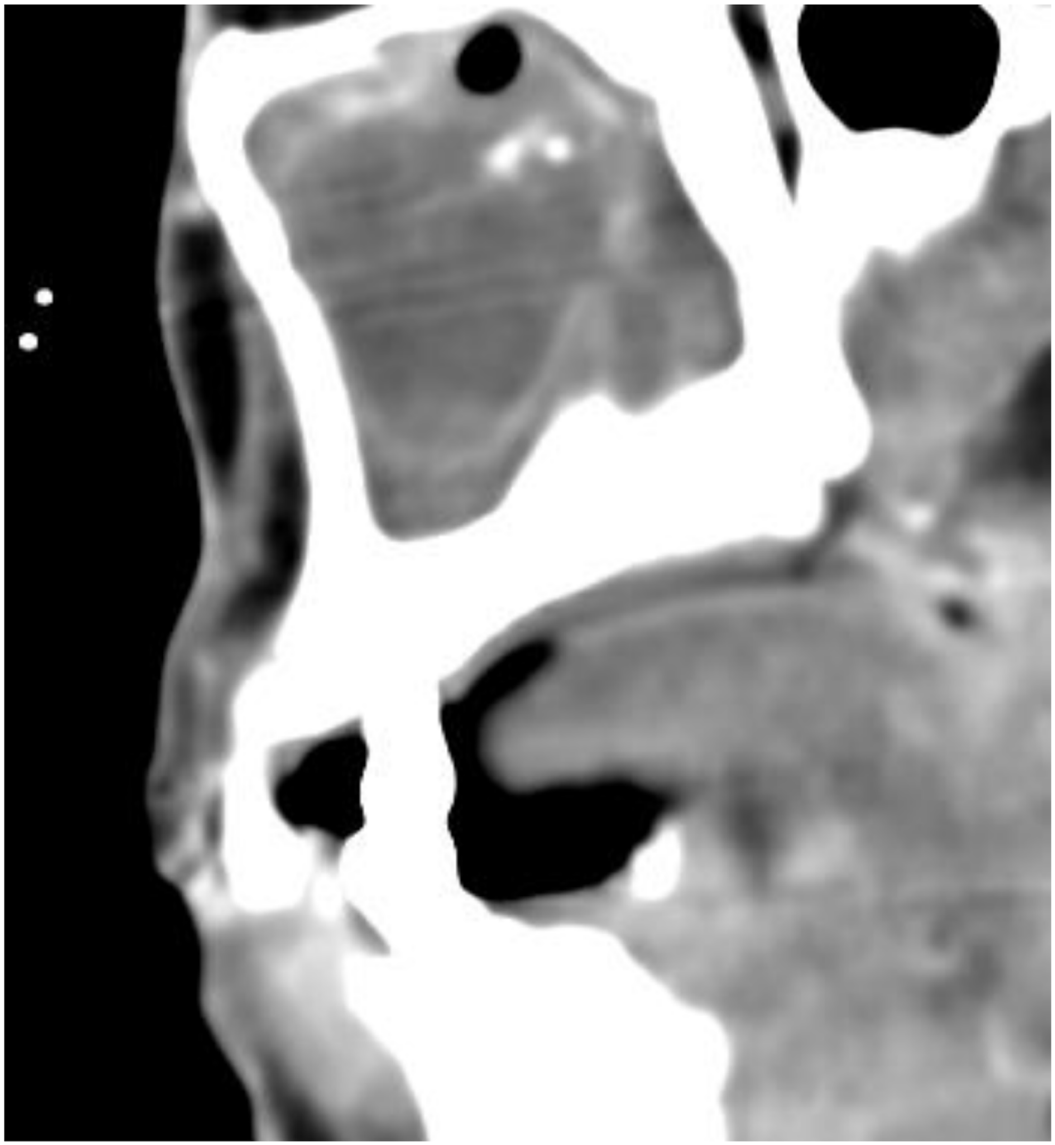
CT-scan in a sagittal section showing a mucosal swelling and obstruction of the maxillary sinus due to an aspergilloma (Contrast medium enhancement).

With regard to the medical history/previous illnesses and symptoms of the patient, an atypical chronic sinusitis should be considered with these indications. Imaging procedures such as computed tomography often show a typical ring-shaped uptake of contrast medium with accompanying polypoid swelling of the sinus´ mucosa in the respective paranasal sinus (Figure 1) [9]. Furthermore in some cases the compressed root filling material can be recognized in the center of the aspergilloma.

The decisive therapy pillars for sinusitis associated with aspergillosis consist of anti-infective therapy (antibiotics, antimycotics), surgical removal of the aspergilloma and inflamed mucosa (osteoplastic maxillary sinus surgery), and treatment of the concomitant disease (e.g., Antidiabetic medication for diabetes mellitus) [10]. In the field of maxillofacial surgery, a transoral access via a vestibular or marginal incision in the area of the facial maxillary sinus wall is particularly suitable in order to carry out the aspergilloma excision and osteoplastic maxillary sinus surgery consecutively via a bone window. In the present article, we were also able to clearly demonstrate that an additional supraturbinal antrostomy with the accompanying insertion of a drain ensures adequate ventilation [8]. Furthermore, microbiological and histopathological tissue processing is often required in order to initiate pathogen-specific therapy after calculated antibiotic/antimycotic therapy.

In addition, it is particularly important to rule out invasive forms of aspergillosis. The therapy of immune-relevant comorbidities requires interdisciplinary treatment. Up to now, there is little data on the long-term results of aspergilloma-associated sinusitis. The follow-up consists of regular clinical examinations as well as additional imaging procedures (MRI, DVT), especially in the case of odontogenically associated aspergilloma infections of the paranasal sinuses.

Fungal infections are a rare cause of sinusitis. A detailed anamnesis and clinical examination of the patient should be carried out, particularly in the case of therapy-refractory forms of chronic sinusitis. It is also important to consider atypical causes and disease connections (root canal treatment, aspergilloma) when dealing with chronic sinusitis. Interdisciplinary diagnostics and therapy are crucial for the successful treatment of this rare entity.
